# Pharmacist intervention in the prevention of heart failure for high-risk elderly patients in the community

**DOI:** 10.1186/s12872-015-0173-3

**Published:** 2015-12-24

**Authors:** Vivian W. Lee, Libby M. Choi, Winki J. Wong, Ho Wing Chung, Carman K. Ng, Franco W. Cheng

**Affiliations:** School of Pharmacy, Faculty of Medicine, The Chinese University of Hong Kong, Shatin, N.T. Hong Kong

**Keywords:** Heart Failure prevention, Pharmacist intervention, Community outreach

## Abstract

**Background:**

Heart failure has become one of the major causes of hospitalization worldwide. Hypertension, diabetes mellitus and hyperlipidemia are the major causes of heart failure. In order to effectively prevent heart failure, blood pressure, blood glucose and cholesterol levels shall be closely monitored and controlled as well as medication adherence. This study aimed to investigate the role of pharmacist intervention in prevention of heart failure in high risk elderly patients in the community of Hong Kong.

**Aim:**

This study aimed to investigate the role of pharmacist intervention in prevention of heart failure in highrisk elderly patients in the community of Hong Kong.

**Methods:**

This prospective uncontrolled study was conducted between July 2012 and April 2013 with two revisits every 3 to 4 months to review elderly patients’ medication profiles as well as assess their blood pressure (BP), random capillary blood glucose (RCBG) level, cholesterol levels, signs and symptoms of heart failure and the compliance level. The results collected at the baseline data were analyzed and compared with those collected at the last follow-up visit.

**Result:**

A significant increase in number of subjects free of symptoms of heart failure (31.88%, *p* < 0.001) was found. For chronic disease management, significant reduction in LDL-cholesterol level (-0.86 ± 0.56mmol/L, *p* = 0.038) and triglyceride level (-1.15 ± 1.09mmol/L, *p* < 0.001) was observed in overall participants. Yet, no significant reduction in BP level or RCBG level was observed in overall subjects. Significant reduction in mean Morisky Medication Adherence Score (-0.54 ± 1.50, *p* = 0.005) indicated improvement in medication compliance in participants.

**Conclusion:**

The Pharmacy Outreach service has a significant role in prevention of heart failure, by means of minimizing heart failure symptoms, improving medication compliance and enhancing chronic disease management, particularly cholesterol management in community elderly patients. This study provided a reference for further investigation and evaluation of the role of pharmacists in preventing heart failure in the high-risk community elderly patients.

**Electronic supplementary material:**

The online version of this article (doi:10.1186/s12872-015-0173-3) contains supplementary material, which is available to authorized users.

## Background

Heart Failure (HF) is a serious condition worldwide, with around 5.7 million people in United States of America being the victims. Also, the disease was found to be a contributing cause in more than 280,000 deaths (1 in 9) in 2008 [1].The American Heart Association has also suggested about half of people who have heart failure die within 5 years of diagnosis [1] and hence the problem should not be neglected. It has been reviewed that the population suffering from heart failure is expanding in Asia, particularly in areas like Hong Kong, due to improved living standards [2]. In Hong Kong, cardiovascular diseases rank the second leading cause of death in age group 65 or above according to the Department of Health [3]. These findings imply primary interventions must be carried out promptly to deal with the problem, in accordance with the Heart Failure Society of America (HFSA) suggestion that early identification and treatment of risk factors are recognized of utmost importance in limiting the public health impact of heart failure [4].

It has been investigated that hypertension [4, 5, 7], diabetes mellitus [4, 5, 6, 7] and hyperlipidemia [4, 6] are the major causes of HF. To effectively prevent HF, blood pressure (BP), blood glucose and cholesterol level shall be closely monitored and controlled. Moreover, medication compliance plays an incumbent role to achieve desirable therapeutic outcomes [7]. It reduces incidents of hospitalization as well as the healthcare cost [8] in community elderly patients, their compliance to medical regimen shall be emphasized to prevent HF. A foreign study has shown that home-based intervention performed by pharmacists could significantly reduce mortality and recurrent hospitalization in HF patients [9]. Meanwhile, local studies suggest that a pharmacy outreach service (POS) for community elderly patients can effectively improve BP control [10] and drug related problems (DRPs) [11]. Besides, it has been illustrated that pharmacists have an established role in managing DRPs in elderly patients, including drug-drug interactions and therapeutic duplications [12]. This study aimed to investigate the role of pharmacist intervention in the early identification of HF symptoms in high-risk elderly patients in the community. We therefore conducted a series of POS visits to perform pharmacist interventions on managing the risk factors of HF in the high-risk community elderly patients, hypertension, diabetes, hyperlipidemia as well as reviewing their medication compliance, for the prevention of HF.

## Methods

This was a prospective uncontrolled study of which subjects were recruited from seven elderly centers in Hong Kong during the Pharmacy Outreach Service in July and August 2012. Patients with recent symptoms of HF were included in this study. These symptoms were evaluated by a questionnaire designed to correspond to the New York Heart Association (NYHA) functional classification of HF. High-risk subjects would also be selected, defined as two out of three of the following criteria were met:

1. High blood pressure as defined in Joint National Committee (JNC) 7 [13]: ≥ 130/80mmHg for diabetic patients and ≥ 140/90mmHg for non-diabetic patients
2. High blood glucose as defined in Standard of Medical Care in Diabetes 2012 [14]: RCBG ≥ 11.1mmol/L
3. Dyslipidemia as defined in ATP III [15]: LDL level ≥ 2.59mmol/L or TG ≥ 2.3mmol/L

Patients who were younger than 65 years old or diagnosed with the following medical conditions, namely malignancies, dementia, psychiatric disorders, Alzheimer’s diseases, Parkinson’s diseases or epilepsy, were excluded in the study. Patients without the need of pharmacological management of their hypertension, diabetes, or dyslipidemia were also excluded. Other exclusion criteria include previous participations in the Chinese University of Hong Kong (CUHK) Outreach Service, current involvement in other clinical trials and the presence of communication barriers. The current project was approved by the Joint Chinese University of Hong Kong-New Territories East Cluster (CUHK-NTEC) Clinical Research Ethics Committee (Reference number: 2014.012). All recruited subjects had been consented prior to joining the current project.

Subjects were revisited twice every 3 to 4 months from October 2012 to November 2012 and from February 2013 to April 2013. At each visit, subjects’ BP, RCBG level and cholesterol level were measured, and their demographic data and medication profiles were reviewed and recorded. The Omron HEM-7011 electronic blood pressure monitor (Omron Healthcare, Kyoto, Japan) was used for measuring the blood pressure. This electronic device had achieved an “A/A” performance classification under the British Hypertension Society criteria and passed the Association for the Advancement of medical instrumentation requirements [16]. The random blood glucose level was measured with Accu-Chek® Performa (Roche Diagnostics, Switzerland). The lipid panel of each subject was measured with a CardioChek® Analyzer (Polumer Technology Systems Inc., USA). By using the battery-operated analyzer, four categories of measurements were collected, namely total cholesterol (TC), high-density lipoprotein (HDL), low-density lipoprotein (LDL) and triglyceride (TG), from fingerstick capillary samples [17]. The test system is intended for in vitro diagnostic use to test whole blood [18].

**Fig. 1:**
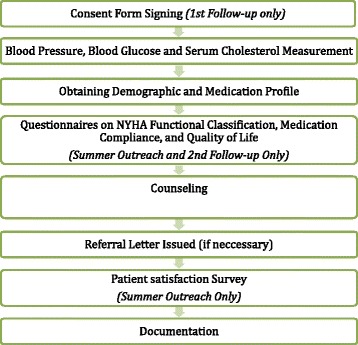
Workflow of each visit

The workflow of each visit is summarized in Fig. 1. Pharmacists counseled on the use of medications, disease knowledge and provided non-pharmacological measures for disease management. Interventions were made with regard to any DRPs identified. Additionally, evaluation on signs and symptoms of HF by a questionnaire (Additional file 1) corresponding to the NYHA functional classification and assessment on medication compliance by the Morisky 8-item Medication Adherence Scale were performed at the first and last visit.

The baseline data were compared to those obtained in the last follow-up visit in order to analyze the impacts of POS on community elders’ management of risk factors associated with HF. McNemar’s test was used for the assessment of HF symptoms while paired t-test was utilized to detect changes in BP, RCBG, cholesterol levels and Morisky Medication Adherence Scale. According to previous POS studies [19–21], mean standard deviation of the change of systolic and diastolic blood pressures are 10.67 and 8.55mmHg respectively.

The number of subject needed was calculated using the equation below [22]:

$$ n=2\left[\frac{\sigma \left({z}_{\alpha }+{z}_{\beta}\right)}{\mu_1-{\mu}_2}\right] $$

At least 40 subjects would be required to identify a 10mmHg change of the systolic blood pressure and at least 26 subjects would be needed to identify a change in 10mmHg of diastolic blood pressure with a power of 80%, a significance level of 0.05 and with an assumption of 40% drop-out rate. Therefore, at least 66 subjects would be required.

All statistical analysis were performed using SPSS 16.0 and a *p* value of <0.05 was defined as statistically significant.

## Results

We screened 297 patients and a total of 103 patients were recruited. The demographic characteristics and medical history of participants is summarized in Table 1. The mean age of participants was 78.19 ± 6.87 years old, and they were taking an average of 5.08 ± 3.02 chronic medications. For the HF symptoms assessment, a significant increase in number of 31.88% subjects (*p* < 0.001) were found to be free of symptoms of HF, spanning the period between baseline and the last visit. A significant reduction of mean number of HF symptoms experienced by each subject was observed (*p* < 0.001). In addition, there were fifteen less elderly patients (-21.74%, *p* = 0.001, Table 2) experienced four or more symptoms at the end of the study. It was observed that more than half (52.17%) of the subjects reported to have limitation of physical activity at baseline and the number was significantly reduced at the end of the study (-26.09%, *p* = 0.001) based on the questionnaire. The other HF symptoms that were found to be sensitive to pharmacist intervention included, shortness of breath (-18.84%, *p* = 0.004), fatigue easily (-17.39%, *p* = 0.029), edema (-17.39, *p* = 0.008) and physical activity limitation when climbing stairs in particular (-18.84%, *p* = 0.015) (Table 2).

**Table 1:** Baseline characteristics of the study participants

Subject characteristics	N = 103 (%)
Age, mean ± SD, years	78.19 ± 6.87
Male sex	31 (29.66)
Literate	83 (80.58)
Live alone	37 (35.92)
Smoking	
Never	80(77.67)
Quitted	19(18.45)
Current	2(1.94)
Unknown	2(1.94)
Exercise Habit	
Amount per week,mean ± SD, minutes	243.43 ± 202,19
- Less than 150 min per day	36(34.95)
− 150 min or above	62 (60.19)
- Unknown	5 (4.85)
Disease Status at baseline visit	
Uncontrolled blood pressure level	57 (55.34)
- Stage II Hypertension^a^	19/57 (33.33)
Uncontrolled RCBG level^b^	20(19.42)
Uncontrolled cholesterol levels	56(54.37)
- Uncontrolled LDL-C level^c^	21/56(37.50)
- Uncontrolled TG level^d^	46/56 (82.14)
- Both uncontrolled LDL-C and TG levels	11/56(19.64)
Present with symptoms of heart failure corresponding to NYIIA Functional Classification system	87 (84.47)
Medical history	
Hypertension	97 (94.17)
Diabetes mellitus	44 (42.72)
Hyperlipidemia	76(73.79)
Coronary Heart Disease	20(19.42)
Heart failure	4(3.88)
Stroke	13(12.62)
Medications	
Number of chronic medications, mean ± SD	5.08 ± 3.02

a: Non-stage II - Systolic blood pressure < 160mmHg Or Diastolic blood pressure < 100mmHg

Stage II - Systolic blood pressure≧160mmHg Or Diastolic blood pressure≧100mmHg (JNC 7 Guideline [13])

b: Uncontrolled RCBG level: ≥ 11.1 mmol/L (ADA [14])

c: Uncontrolled LDL-C level: ≥ 2.59 mmol/L (ATP III [15])

d: Uncontrolled TG level : ≥ 2.3mmol/L (ATP III [15])

**Table 2:** Summary of various heart failure symptoms experienced by subjects based on the heart Failure Symptoms Assessing Questionnaire at baseline and latest follow-up visit, N = 69

Question no.	Symptoms of heart failure	Baseline n(%)	Latest visit, n(%)	Difference, n(%)	*P* value
1	Chest pain	22 (31.88)	13(18.84)	−9 (13.04)	0.064
2	Shortness of breath	22 (31.88)	9(13.04)	−13 (18.84)	**0.004**
3	Dyspnea	10 (14.49)	5 (7.25)	−5(7.25)	0.125
4	Dizziness or fainting when position changed	18 (26.09)	10 (14.49)	-8 (11.59)	0.057
5	Dyspnea when. lying down	3 (4.35)	5 (7.25)	+2 (2.90)	0.727
6	Fatigue easily	35 (50.72)	23 (33.33)	−12 (17.39)	**0.029**
7	Edema	22 (33.33)	11 (15.94)	−12(17.39)	**0.008**
8	Sleeping pattern disturbed due to difficulty breathing	10 (14.49)	7(10.14)	−3 (4.35)	0.549
9	Idiopathic cough	10 (14.49)	4 (5.80)	−6(8.70)	0.109
10	Limitation of physical activity	36 (52.17)	18 (26.09)	−18 (26.09)	**0.001**
10a	- Discomfort when climbing stairs	31 (49.28)	18 (26.09)	−13 (18.84)	**0.015**
10b	- Discomfort when walking	9(13.04)	5 (7.25)	-4 (5.80)	0.344
10c	- Discomfort at rest	2 (2.90)	5 (7.25)	+3 (4.35)	0.375
Total no. of subject symptoms of heart failure		63 (91.30)	41 (59.42)	−22 (31.88)	**<0.001**
No. of symptoms experienced by individual, mean ± SD		2.96 ± 1.93	1.52 ± 1.91	−1.43 ± 1.90	**<0.001**
Symptoms experienced by most subjects		Limitation of physical activity	Fatigue easily		
2^nd^ most symptoms experienced by subjects		Fatigue easily	Limitation of physical activity		
No. of subjects experienced					
1 symptom		10 (14.49)	15(21.74)	+5 (7.25)	0.225
2 symptoms		14 (3.0.29)	9(13.04)	−5(7.25)	0.251
3 symptoms		15 (21.74)	8 (11.59)	−7 (10.14)	0.108
≥4 symptoms		24 (34.73)	9(13.04)	−15(21.74)	**0.001**

For the impact of POS on the risk factors management of heart failure, namely hypertension, diabetic mellitus and hyperlipidemia, no significant improvement was observed in BP and glycemic control in overall participants. However, significant reduction in SBP level (-14.45 ± 17.97mmHg, *p* = 0.024) was achieved in subjects with Stage II hypertension. The reduction of RCBG level (-3.70 ± 2.44mmol/L, *p* = 0.004) was also significant in not well-controlled subjects (Table 3). For lipid control, significant improvement was achieved in overall participants, with significant reduction in LDL-cholesterol level (-0.86 ± 0.56mmol/L, *p* = 0.038), and in triglyceride level (-1.15 ± 1.09mmol/L, *p* < 0.001).

**Table 3:** Comparison of blood pressure, random capillary blood glucose and cholesterol level between baseline and the latest follow-up visit

Systolic blood pressure (SBP) level (mean ± SD, mmHg)					
	N	Baseline	Latest	Difference	*P* value
All	61	142.11 ± 19.73	145.00 ± 19.37	+2.89 ± 22.02	0.310
Uncontrolled subject^a^	11	169.27 ± 11.59	154.82 ± 16.32	−14.45 ± 17.97	**0.024**
Diastolic blood pressure (DBP) level (mean ± SD, mmHg)					
All	61	70.20 ± 10.70	71.87 ± 9.54	+1.67 ± 10.35	0.256
Uncontrolled subject^a^	11	71.00 ± 9.96	72.45 ± 11.24	+1.24 ± 13.49	0.728
Random capillary blood glucose (RCBG) level (mean ± SD, mmol/L)					
All	61	7.81 ± 2.79	7.78 ± 2.89	−0.03 ± 2.96	0.938
Uncontrolled subject^b^	8	13.45 ± 2.57	9.75 ± 3.63	−3.70 ± 2.44	**0.004**
LDL-cholesterol level (mean - SD, mmol/L)					
All	57	2.23 ± 0.91	2.05 ± 0.57	−0.19 ± 0.66	**0.038**
Uncontrolled subject^c^	17	3.42 ± 0.54	2.56 ± 0.46	−0.86 ± 0.56	**<0.001**
Triglyceride level (mean ± SD, mmol/L)					
All	60	2.40 ± 1.11	1.71 ± 0.90	−0.68 ± 1.01	**<0.001**
Uncontrolled subject^d^	30	3.28 ± 0.86	2.13 ± 1.00	−1.15 ± 1.09	**<0.001**

a: uncontrolled subjects with SBP/DBP >140/90 mmHg or >130/80 mmHg (DM patients) (JNC 7 [13])

b: uncontrolled subjects” with baseline RCBG > 11.1 mmol/L (ADA [14])

c: uncontrolled subjects with LDL-level > 2.59 mmol/L(ATP III [15])

d: uncontrolled subjects with TG-level >2.3 mmol/L(ATP III [15])

Non-compliance to medications is also a contributing factor of HF, 66.99% of the elderly patients were identified to be medication non-compliant. The Morisky score results showed a significant reduction in mean score by 0.54 ± 1.50 (*p* = 0.005) (Table 4). At the baseline, 27.54% of the subjects were considered as low compliance subjects and there was a slight but insignificant reduction in the number at the end of the study (-4.35%, *p* = 0.607). In addition, results showed a significant increase by 23.19% (*p* = 0.001) in the number of subjects with high compliance level. Forgetfulness (30.4%) was found to be the most common reason for non-compliance, followed by improper administration time (20.3%) and fear of adverse drug reaction (18.8%).

**Table 4:** Identified causes of non-compliance and the compliance score at Baseline and the latest Follow-up visit, N = 69

Causes of non-compliance	N (%)			
Forgetfulness	21/69 (30.43)			
Dosage adjustment	8/69(11.59)			
Frequency adjustment	8/69(11.59)			
Improper administration time	14/69 (20.29)			
Improper administration method	3/69 (4.35)			
Overuse of “PRN” drugs	3/69 (4.35)			
Underuse of “PRN” drugs	1/69(1.45)			
Fear of Adverse drug reaction	13/69(18.84)			
Think no need	7/69(10.14)			
Pill splitting method	2/69 (2.9)			
	Compliance score^J^			
	Baseline	Latest	Difference	*p* value
mean ± SD	1.93(±1.78)	1.39(±1.80)	−0.54(±1.50)	**0.005**
No. of Low adherence subject^k^,n(%)	19 (27.54)	16(23.19)	−3 (4.35)	0.607
No. of Medium adherence subject^k^’, n (%)	33 (47.83)	20 (28.99)	−13(18.84)	**0.011**
No. of High adherence subject^k^, n (%)	17(24.64)	33 (47.83)	+16(23.19)	**0.001**

J: Compliance based on Morisky questionnaire

k: Low adherence - Morisky Scores >2; Medium adherence - Morisky Scores = 1 or 2; High adherence - Morisky Scores = 0

## Discussion

It has been suggested by HFSA that patients at risk of cardiovascular disease are also at high risk for developing HF, and early identification and treatment of risk factors are recognized of utmost importance in limiting the public health impact of HF [4]. Hypertension, hyperlipidemia, diabetes mellitus and unhealthy lifestyle are recognized as potential risk factors for the development of myocardial remodeling, cardiac dysfunction and hence HF. In the study, subjects were being assessed with the presence of HF symptoms using the HF Symptoms Assessment Questionnaire via self-reporting method. There were a total of 16 questions in the questionnaire that correspond to ten symptoms of HF. Significant reduction in number of HF symptoms experienced by each subject as well as number of subjects experienced four or more symptoms were observed, which demonstrated the changes before and after the pharmacists’ interventions. Although the changes may not be solely due to the pharmacists’ intervention due to the lack of control group in the current study. It demonstrated the potential essential role of pharmacist intervention upon better disease control and minimizing risk of HF in community elderly patients. These results correlate to a previous study suggesting that pharmacists have an incumbent role in the community in HF management [16].

In addition, the role of pharmacist in managing risk factors of HF was also investigated in the study. According to the US NHANES I Epidemiologic Follow-up Study, not well-controlled hypertension was found to be positively and significantly associated to increased risk of HF [17]. This study has shown no significant improvement in the BP management in overall participants, however in subgroup analysis, statistically significant reduction in SBP level was detected in participants with stage-II hypertension at baseline. This result is consistent with a previous study which has shown that hypertensive patients with baseline SBP ≥160mmHg would have a more significant decrease in BP than the others who have lower BP baseline after pharmacist intervention [25]. Therefore, hypertensive patients, who are of higher risk to developing HF, are more sensitive to pharmacist intervention in the prevention of HF.

It was clearly established in prior studies that diabetes is a risk factor for HF [26-29]. In the study, no significant improvement in glycemic control was observed in overall participants. The reason for the insignificant result might be due to the fact that RCBG level was highly subjected to the influence of various confounding factors, such as time elapsed after food intake. As a result, RCBG level might not able to truly reflect the impact of pharmacist intervention on glycemic control in overall participants. Improvement in study design, such as measuring HbA1c level instead of RCBG level, is suggested in future investigation.

HFSA has suggested that hyperlipidemia is a risk factor for the development of HF [4]. Meanwhile, since hyperlipidemia is one of the risk factors for the development of CHD [30], well control of cholesterol levels, particularly LDL-cholesterol level, can reduce risk of CHD and subsequently myocardial remodeling and heart failure. This study has showed that pharmacist intervention significantly reduce LDL-cholesterol level as well as triglyceride level in overall participants. In the sub-group analysis, subjects with not well-controlled cholesterol levels were found to be more sensitive to pharmacist intervention in managing hyperlipidemia. Precedent studies have found that regular pharmacist follow-up and intervention for patients on disease education, cholesterol measurement, medication compliance and referral to physician can improve cholesterol management in high-risk patients [31, 32]. Systemic review also showed that community pharmacy-based service contributed to the reduction in risk factors for CHD regarding lipid management [17]. Therefore, pharmacist intervention in POS may contribute in prevention HF in community elderly patients but further investigation shall be conducted to provide solid evidence on this issue.

Several studies have suggested that non-compliance can be a precipitating factor of HF exacerbation [18, 33] as well as impairing the chronic disease management [34]. Pharmacist therefore are in ideal position to evaluate patients’ compliance and aid them to improve their medication compliance. Therefore, it is essential for pharmacist to ensure patients with chronic diseases are compliance with medications to prevent HF or its progression. In this study, the Morisky score results showed a significant improvement in medication compliance among subjects with pharmacist intervention, which provide medical education on individual’s regimen. Over 66% of subjects were identified to have non-compliance issue, which is one of the well-known common DRPs in elderly patients [35]. Out of the various reasons behind the non-compliance issue, forgetfulness contributed the most (30.4%). Forgetfulness is classified as an unintentional cause of non-compliance. Previous study [36] illustrates the importance of pharmacist’s intervention in providing memory aids, educating measures to take if a dose is missed and suggesting the use of medication compliance calendars. Therefore, additional compliance aids and education shall be provided in the future POS.

### Limitations

The current study has several limitations. There was selection bias during sampling since this was not a randomized trial, which was also reflected in the unequal distribution in gender in the sample. There were more female subjects involved in the POS probably due to their better health-seeking behavior but this was consistent with the previous POS [10]. A randomized controlled trial with cross-over design might be considered not only to reduce the selection bias but also to provide a more concrete evidence for the benefits of pharmacist interventions. Furthermore, only seven elderly centers participated in this study and this may not be truly reflecting the situation in Hong Kong. We did not assess the echocardiography results to confirm the impact of POS. The compliance assessment was based on self-reporting from the patients. The current HF questionnaire developed for this study was not validated and therefore may not be applicable for patients outside Hong Kong. Not all risk factors were examined in this study. The sample size of the current study was small and Hong Kong-based. As a result, the study may be underpowered and not be able to generalize to other countries with a larger population.

## Conclusion

Pharmacy outreach service has significantly improved HF symptoms management and medication compliance in the high-risk community elderly patients. It has also improved cholesterol management in overall subjects. Further investigation with improved study design shall be conducted in order to evaluate and provide sound evidence on pharmacist role in preventing HF in community elderly patients.

### Ethical approval

The study was approved by the Ethics Committee, CUHK-New Territories East Cluster, Hospital Authority, Hong Kong.

## Additional file

Additional file 1:
**Appendix 1 - Heart Failure Symptoms AssessingQuestionnaire.** (DOCX 27 kb)

## Abbreviations

BP: Blood Pressure
CUHK: Chinese University of Hong Kong
CUHK-NTEC: Chinese University of Hong Kong-New Territories East Cluster
DRPs: Drug-related problems
HF: Heart Failure
HFSA: Heart Failure Society of America
HDL: High Density Lipoprotein
IFCC: International Federation of Clinical Chemistry and Laboratory Medicine
JNC: Joint National Committee
LDL: Low Density Lipoprotein
NYHA: New York Heart Association
POS: Pharmacy Outreach Service
RCBG: Random Capillary Blood Glucose
TC: Total Cholesterol
TG: Triglycerides
