# Supplementary material for: Pharmacist intervention in the prevention of heart failure for high-risk elderly patients in the community
Source: BMC Cardiovasc Disord. 2015 Dec 24;15:178. doi: 10.1186/s12872-015-0173-3 (PMC4690278; doi:10.1186/s12872-015-0173-3)
Supplement: Additional file 1: — Appendix 1 - Heart Failure Symptoms AssessingQuestionnaire. (DOCX 27 kb) [file 12872_2015_173_MOESM1_ESM.docx]

**Additional file 1 - Heart Failure Symptoms Assessing Questionnaire**

New York Heart Association Functional Classification

1. Do you experience chest pain recently?

☐Yes ☐No

2. Do you experience short of breath recently?

☐Yes ☐No

3. Do you ever feel difficult to breathe recently?

☐Yes ☐No

4. Do you feel dizzy when your body position is changed?

☐Yes ☐No

5. Do you feel hard to breathe when you lie down?

☐Yes (Please continue) ☐No (Please go to Question 6)

(a) Will it be improved when you lay bolstered up?

☐Yes ☐No

(b) About how many cushion(s) will make you feel comfortable? pillow(s)

6. Do you easily get tired?

☐Yes ☐No

7. Do you ever have edema before?

☐Yes ☐No

8. Do breathing problem affect your sleeping quality?

☐Yes ☐No

9. Do you often cough?

☐Yes (Please continue) ☐No (Please go to Question 10)

(a) Do you know the reason of coughing?

☐Yes, it is because ☐No

10. Mobility

(a) Do you feel unwell when you get upstairs?
 (E.g. feel exhausted, difficult to breathe, short of breath)

☐Yes ☐No

(b) Do you feel unwell when you walk on a plain path?

☐Yes ☐No

(c) Do you feel unwell when you are at rest (like sitting down)?

☐Yes ☐No
